# Microwave-assisted enzymatic hydrolysis to produce xylooligosaccharides from rice husk alkali-soluble arabinoxylan

**DOI:** 10.1038/s41598-021-03360-2

**Published:** 2022-01-07

**Authors:** Wannaporn Klangpetch, Alisa Pattarapisitporn, Suphat Phongthai, Niramon Utama-ang, Thunnop Laokuldilok, Pipat Tangjaidee, Tri Indrarini Wirjantoro, Pannapapol Jaichakan

**Affiliations:** 1 Faculty of Agro-Industry, Chiang Mai University, Chiang Mai, 50100 Thailand; 2 Cluster of High Value Products from Thai Rice and Plants for Health, Chiang Mai University, Chiang Mai, 50100 Thailand; 3 Cluster of Innovative Food and Agro-Industry, Chiang Mai University, Chiang Mai, 50100 Thailand; 4 Graduate School of Agriculture, Saga University, Honjo, Saga, 840-8502 Japan; 5 Research Center for Development of Local Lanna Rice and Rice Products, Chiang Mai University, Chiang Mai, 50200 Thailand; 6 Department of Agro-Industry, Faculty of Agriculture Natural Resources and Environment, Naresuan University, Phitsanulok, 65000 Thailand

**Keywords:** Biotechnology, Microbiology

## Abstract

The prebiotic properties of xylooligosaccharides (XOS) and arabino-xylooligosaccharides (AXOS) produced from rice husk (RH) using microwave treatment combined with enzymatic hydrolysis were evaluated. The RH was subjected to microwave pretreatment at 140, 160 and 180 °C for 5, 10 and 15 min to obtain crude arabinoxylan (AX). Increasing microwave pretreatment time increased sugar content. Crude AX was extracted with 2% (w/v) sodium hydroxide at 25 °C for 24 h and used as a substrate for XOS production by commercial xylanases. Results showed that oligosaccharides produced by Pentopan Mono BG and Ultraflo Max provided xylobiose and xylotriose as the main products. AXOS was also present in the oligosaccharides that promoted growth of *Lactobacillus* spp. and resisted degradation by over 70% after exposure to simulated human digestion.

## Introduction

The rice husk (RH) acts as a sheath to protect the rice seed during the growth period. The rice milling process generates approximately 28% of RH as a by-product that is mostly used as a solid fuel. Previous studies reported that RH contains lignocellulosic material that consists of cellulose and hemicellulose at 36% and 29%, respectively^1^. The hemicellulose structure of RH contains long-chain xylose units as the backbone, called xylan. Xylan comprises a group of hemicelluloses, that are composed of β-1,4-linked xylose residues with or without side branches of acetyl groups, arabinose, glucuronic acid and 4-O-methyl glucuronic acid^2^. Arabinoxylans (AX) are one of the four types of xylans, that have a backbone of β-(1,4)-linked xylose residues, substituted with arabinose residues at the C(O)-2 and/or C(O)-3 positions^3^.

Conversion of AX by enzymatic hydrolysis into xylooligosaccharides (XOS) and arabino-xylooligosaccharides (AXOS) has gained considerable interest because of the mild conditions used and the specific products obtained. XOS have more effective properties than other oligosaccharides. Recent reports indicated that fermentation of XOS by *L. brevis* S27, *L. pentosus* S42 and *L. plantarum* S61, with enhanced antifungal and antibacterial activities compared to fructo-oligosaccharides (FOS)^4^. The effective daily dose of oligosaccharides indicating that XOS has a greater range of health benefits than FOS and inulin at a lower dose^5^. XOS are also the only nutraceutical that can be obtained from lignocellulosic biomass as a low-cost and abundantly available raw material^6^. XOS are non-digestible food ingredients with prebiotic properties for selectively promoting the growth of probiotics, thereby providing many health benefits and several applications on food and pharmaceutical industries^7^. Growth of *Bifidobacterium breve*, *B. lactis*, *B. adolescentis*, and *B. bifidum* has been demonstrated to ferment XOS in vitro and in vivo^8^. Production of XOS and AXOS is usually performed by endo-1,4-β-D-xylanase (EC 3.2.1.8), which is the major enzyme involved in the breakdown of β-1,4-linked xylan into XOS of varying lengths and has tolerance for arabinose substitutions in the xylan chain^9^. The activities of exo-1,4-xylosidase (EC 3.2.1.37) and exo-1,3-xylosidase (EC 3.2.1.72) are key in the hydrolysis of xylan to xylose^10^. Xylanases with different activities and substrate specificities produce various end products in enzymatic hydrolysis^11^. This research focused on two xylanases belonging to the glycoside hydrolase families 10 and 11. GH11 xylanases preferentially cleave unsubstituted regions of the AX backbone and whereas GH10 enzymes are less hampered by the presence of substituents along the xylan backbone, cleave the decorated regions^12^. Therefore, differences in substrate specificity have important implications in the deconstruction of xylan in biomass^13^.

Recently, many techniques have been applied for AX extraction. Fermentation or solvent extraction can reduce the loss of important substances compared to the heating process. Acid detergent fiber, neutral detergent fiber, hemicellulose, and crude cellulose of lignocelluloses were lost in biomass treated with fungal fermentation^14^. However, these methods are time-consuming and produce various chemical wastes. Microwave extraction may overcome these disadvantages with short processing time and low amount of solvent utilization^15^. Microwave pretreatment is a promising technology for biomass conversion, with efficiency dependent on the dielectric properties of the biomass and solvent, allowing the material to be retained. The electromagnetic power is converted into heat, causing lignocellulosic material breakdown via molecular collision due to dielectric polarization^16,17^. Other heating processes such as hydrothermal treatment using high temperatures for a longer time, result in the loss of important substances including large amounts of monosaccharides. Gissibl et al.^18^ recently revealed that microwave pretreatment at 170 °C for 2 min enhanced enzymatic production of soluble β-1,3-glucans, while microwave treatment at 200 °C for 5 min significantly increased oligosaccharide extraction yield from spruce wood^19^. Coelho et al.^20^ reported that microwave superheated water at 210 °C with dilute alkali recovered 43% of AX and AXOS from spent grain after the brewing process, while Kundu et al.^21^ stated that cleavage of alkali-labile linkages between hemicellulose and other associated constituents depended on its natural performance.

A prebiotic can be defined as “a substrate that is selectively utilized by host microorganisms conferring a health benefit. This definition expands the concept of prebiotics to possibly include non-carbohydrate substances, applications to body sites other than the gastrointestinal tract, and diverse categories other than food” (Gibson et al., 2017). AX can be transformed into XOS and AXOS that are considered to be functional foods due to their potential prebiotic properties. XOS and AXOS enter the colon intact and serve as carbon sources for bacteria including *Bifidobacterium* and *Lactobacillus*^22^. Probiotic fermentation assists the development of short-chain fatty acids such as acetate, propionate and butyrate, which provide the host with metabolic energy and intestinal acidification^23^.

This study investigated the effects of temperature and microwave pretreatment time on RH-derived AX extraction to produce XOS and AXOS by GH10 and GH11 xylanases. The growth promotion of lactic acid bacteria and digestion resistance were evaluated using an in vitro human digestion simulation to demonstrate the prebiotic potential of XOS and AXOS. This research will maximize the use of rice husk by-product as an alternative prebiotic source.

## Materials and methods

### Materials

Rice husk (RH) was kindly provided by a local rice milling plant in Phitsanulok Province, Thailand. The RH was dried in a hot-air oven at 40 °C until the moisture content was lower than 10% (w/w), then crushed with a blender and sieved through a 40 µm mesh screen. RH powder was stored in a zip lock bag at 30 °C. The method of the Association of Official Analytical Chemists was used for determination of moisture, crude fiber, protein, fat, ash and carbohydrate of RH^24^.

Commercial xylanases as Ultraflo Max (700 U/mL from *Aspergillus oryzae* and *Trichoderma reesei*) and Pentopan Mono BG (2,500 U/g from *Thermomyces lanuginosus*) were purchased from Novozyme Co., Ltd., Denmark. All chemicals and solvents were of analytical grade. Xylose (Merck, Germany), arabinose (Sigma, Germany), mannose (Merck, Germany), galactose (Sigma, Germany) and glucose (Sigma, Germany) were used as standards for determination of carbohydrate composition. A mixture of arabinose (A1) (Sigma, USA), xylose (X1) (Merck, Germany), xylobiose (X2), xylotriose (X3), xylotetraose (X4), xylopentaose (X5), xylohexaose (X6), (Wako, Japan), 2^3^-α-L-arabinofuranosyl-xylotriose (A2XX), 3^2^-α-L-arabinofuranosyl-xylobiose (A3X), 3^3^-α-L-arabinofuranosyl-xylotetraose (XA3XX) and 2^3^,3^3^-di-α-L-arabinofuranosyl-xylotriose (A2,3XX) (Megazyme, Ireland) were used as standards for the determination of oligosaccharides. Commercial prebiotics were used to compare prebiotic properties with the obtained oligosaccharides. A 95% commercial XOS (XOS95P) was purchased from AWBIO, Taiwan, while resistant maltodextrin (RMD) and inulin were purchased from Blenntag Ingredients, Thailand. Simulated human digestion enzymes, α-amylase from *A. oryzae* (40,000 U/mL), pepsin from porcine gastric mucosa (3200 U/mg) and pancreatin from porcine pancreas (8X USP) (Sigma, Germany) were used as simulated human digestion enzymes.

### Pretreatment of RH

One gram of previously prepared RH powder was soaked in 20 mL of acetone and ethanol mixture in the ratio of 1:2 (v/v) at 30 °C for 24 h. The RH residue was filtered through Whatman No. 1 filter paper and washed with boiling water. After rewashing with distilled water, the residue was dried at 45 °C for 24 h in a hot air oven to obtain extractive-free RH, and used in microwave pretreatment.

### Determination of carbohydrate composition

The determination methods for structural carbohydrates of RH and extractive-free RH were modified from Jaichakan et al.^25^. Briefly, 0.4 g of extractive-free RH was pre-hydrolysed with 4.5 mL of 72% sulfuric acid and mixed for 30 min in a mortar. Upon completion of pre-hydrolysis, the slurry was diluted to a final acid concentration of 4% by adding 84 mL distilled water and autoclaved for 1 h at 121 °C. After completion of the autoclave cycle, a 10 mL aliquot was transferred and neutralized to pH 5–6 with calcium carbonate. This aliquot was used to determine structural carbohydrates by high-performance anion exchange chromatography with pulsed amperometric detection (HPAEC-PAD, Dionex ICS-5000 Ion Chromatography, Thermo Scientific, Bellefonte PA, USA) with a Dionex CarboPac PA-1 column (250 mm × 4 mm) and a guard column (50 mm × 4 mm) with an injection volume of 20 µL and a flow rate of 1.0 mL/min. The post-column pump operated at a flow rate of 0.5 mL/min with 300 mM sodium hydroxide. A stepwise linear gradient was applied over 20 min with 100% distilled water and applied over 16 min by mixing solutions of 200 mM sodium hydroxide and 200 mM sodium acetate in 170 mM sodium acetate. Eluted monosaccharides were monitored by PAD detection using a gold electrode. A mixture of xylose, arabinose, mannose, galactose, and glucose was utilized at a concentration range of 0–5 ppm as a calibration standard.

Carbohydrate profiles of oligosaccharides produced from RH were determined by HPAEC with a Dionex CarboPac PA-200 column (250 mm × 4 mm) and a guard column (50 mm × 4 mm) with an injection volume of 20 µL and constant flow rate of 0.4 mL/min. The gradient elution program for the sample pump of the neutral carbohydrate was performed as described by McCleary et al.^26^. Oligosaccharides were identified using A1, X1-X6, A2XX, A3X, XA3XX and A2,3XX at a concentration range of 0–5 ppm as standards.

The polymeric sugars were calculated from the concentration of the corresponding monomeric sugars according to the National Renewable Energy Laboratory. For C-5 sugars (xylose and arabinose), an anhydro correction factor of 0.88 was used, with 0.90 for C-6 sugars (glucose, galactose and mannose)^27^.

$${\text{Polymeric}}\;{\text{sugar}} = {\text{monomeric}}\;{\text{sugar}}\;{\text{content}} \times Anhydro\;correction$$

### Microwave pretreatment of extractive-free RH

A closed-vessel microwave digestion system equipped with a 10 position rotor and capable of delivering 1600 W of power (ETHOS 1600, Milestone Inc., Sorisole, Italy) was used for sample pretreatment. The temperature of all samples was directly controlled by this easyTEMP contactless sensor, using a high-pressure and high-temperature rotor (SK-15 easyTEMP high pressure rotor, Milestone Inc., Sorisole, Italy) with a capacity of up to 15 vessels. Vessels used with this machine were made of modified polytetrafluoroethylene (PTFE) with 100 mL volume. Each vessel contained 45 mL of sample. During each run, 15 vessels were fitted in all positions. An aliquot of 1.5 g of the extractive-free RH was suspended in 45 mL of distilled water in the PTFE closed vessel. The heating program was performed at microwave irradiation power of 600, 1100 and 1600 W to reach 140, 160 and 180 °C, respectively in 5 min (come up time), and then held for predetermined times of 5, 10 and 15 min for each temperature. Temperature and pressure sensors were used in all treatments. After completion, the reactant was immediately cooled to 25 °C in a cold water bath. Microwave-pretreated residues were separated by vacuum filtration. The pretreated RH residue was washed with 95% ethanol, then twice with distilled water and dried overnight at 45 °C in a hot air oven to obtain RH water-unextractable AX (RH-WUAX). This was then used to determine the structural carbohydrates as described above, as well as to prepare for the RH alkali-soluble AX (RH-AX) extraction.

### Microstructural analysis of RH after microwave pretreatment

A scanning electron microscope (SEM, EDS 6610LV, JEOL Ltd., Tokyo, Japan) was used to observe the surface morphology of the microwave-pretreated RH. The samples were dried at 65 °C for 24 h in a hot air oven, then ground and passed through a 40 mesh sieve. The dried samples were mounted on aluminum, coated with gold, and then viewed at an accelerating voltage of 15 kV with magnification of 200 to 1000. The diameter of the final beam spot on the sample was 40 nm.

### Extraction of alkali-soluble RH-AX

RH-WUAX was suspended in 2% (w/v) sodium hydroxide (1 g: 25 mL) at 30 °C with continuous shaking at 180 rpm for 24 h. Subsequently, the alkali-soluble AX liquor was collected by centrifugation at 9000 rpm and 25 °C for 15 min. The liquor was adjusted to pH 6.0 with 37% hydrochloric acid, and then 95% ethanol was added to a final ethanol concentration of 80% (v/v) to precipitate RH-AX. The precipitate was centrifuged at 9000 rpm at 4 °C for 10 min, washed with acetone and dried for 24 h at 45 °C in a hot air oven to obtain crude RH-AX. The total sugar content was determined using the phenol–sulfuric method, using glucose as a standard^28^. The reducing sugar content was determined using the dinitrosalicylic acid method (DNS), using xylose as a standard^29^.

### Enzymatic hydrolysis of XOS and AXOS production from RH-AX

Hydrolysis of RH-AX was performed using the two commercial xylanases Pentopan Mono BG and Ultraflo Max. Briefly, 2% (w/v) RH-AX was suspended in 100 mM sodium phosphate buffer at pH 5.0. Then, each xylanase was separately added at enzyme concentrations of 50, 150 and 300 U/g substrate, and incubated at 50 °C in a water bath shaker at 170 rpm for 24 h. Samples were periodically taken at pre-set times and the reaction was stopped by boiling for 5 min. The samples were then dried with a freeze dryer (FreeZone 18 Liter Console Freeze Dryer, Labconco Corp., USA) to obtain an RH oligosaccharide (RH-XOS). Total reducing sugar content was measured by the DNS method, and the carbohydrate profile was screened by thin layer chromatography (TLC). The XOS composition was qualitatively checked by TLC following the method of Jaichakan et al.^30^. In brief, TLC silica gel 60 (Merck, Germany) was used as the stationary phase. The mobile phase consisted of an n-butanol:acetic acid:water solution in a ratio of 2:1:1 by volume. The TLC sheet was sprayed with 10% sulfuric acid in ethanol solution containing 0.2% orcinol. The bands were developed once by heating in a hot air oven at 110 °C. The mixed XOS (X1-X6) (Wako, Japan) was used as the standard, while HPAEC was used to identify the profiles of XOS and AXOS.

Xylanase activity was determined by incubating 50 µL of the diluted enzyme with 50 µL of 1% birch wood xylan in 0.1 M sodium phosphate buffer at pH 6.5, 50 °C. After 10 min, the reaction was stopped by adding 300 µl of DNS reagent. Color was developed by boiling the reaction in water for 10 min, and the process was immediately stopped by placing the reaction tube in a cold water bath. The absorbance was measured at 540 nm and compared to a xylose standard (0–1.0 mg/mL) after 600 µL of distilled water were added and mixed well.

One unit of xylanase activity (U) was defined as the amount of enzyme liberating one µmol of reducing sugar equivalents from 1% birch wood xylan per min at 50 °C.

### Lactic acid bacterial growth promotion of RH-XOS

The RH-XOS and commercial prebiotic utilization of lactic acid strains were evaluated in 96-well microplates. *Lactobacillus plantarum* JCM1149T, *Lactobacillus sakei* JCM1157, and *Lactobacillus bulgalicus* JCM1002 were purchased from the Japan Collection of Microorganisms. *Lactobacillus brevis* TISTR860 was purchased from the Thailand Institute of Scientific and Technological Research (Bangkok, Thailand). *Lactobacillus johnsonii* KUNN19-2, *Lactobacillus reuteri* KUB-AC5, and *Lactococcus lactis* KA-FF-1-4 were obtained from Kasetsart University, Thailand. The strain KUNN19-2 was isolated from Thai-style fermented pork, KUB-AC5 was isolated from chicken intestine and KA-FF-1-4 was isolated from fermented fish^31–33^. The strains were pre-cultured in De Man, Rogosa and Sharpe (MRS) broth at 37 °C for 18 h. MRS broth was reconstituted without glucose according to Nakphaichit et al.^34^ as follows: peptone 1%, beef extract 1%, yeast extract 0.5%, dipotassium hydrogen phosphate 0.2%, sodium acetate, ammonium monohydrogen citrate 0.2%, magnesium sulfate 0.01%, manganese sulfate 0.005% (w/v) and Tween 80 0.1% (v/v). The initial pH of the medium was adjusted to 6.5 by 1 M sodium hydroxide or 1 M hydrochloric acid and autoclaved at 121 °C for 15 min. Xylose, XOS95P, RMD, inulin and glucose were dissolved in modified MRS, and filtered through a sterile 0.2 µm filter 0.2 µm and added to the media to a final concentration of 2% (w/v), whereas RH-XOS was used with 2% reducing sugar content. The microplates were inoculated with cultured lactic acid bacteria at a final concentration of 1 × 10^4^ CFU/mL, and sterile sealing tape was used to prevent vaporization and contamination. The samples were incubated at 37 °C for 24 h. Growth parameters were monitored using a microplate reader (UV–Vis SpectraMax 190 Microplate Reader, Molecular Devices, Sunnyvale, CA, USA) at 600 nm.

### Continuous in vitro digestion of RH–XOS

The method of in vitro digestion followed Minekus et al.^35^. Briefly, RH-XOS, XOS95P, RMD and inulin were added into the simulated salivary fluid (SSF), simulated gastric fluid (SGF) and simulated intestinal fluid (SIF) to study the sugar release. The samples were first added with SSF, and two percentages of sample solutions were mixed with SSF electrolyte stock solution at a ratio of 50:50 (v/v). Amylase solution was added to achieve 75 U/mL in the final mixture at pH 7.0 and incubated for 5 min. The oral bolus sample was mixed with SGF electrolyte stock solution at a final ratio of 50:50 (v/v). Porcine pepsin was added to achieve 2000 U/mL in the final mixture at pH 3.0 and incubated for 2 h. Lastly, gastric chyme was mixed with SIF electrolyte stock solution at a final ratio of 50:50 (v/v). Pancreatin solution was added to achieve 200 U/mL in the final mixture at pH 7.0 and incubated for 2 h. Bile salts were added to give a final concentration of 10 mM in the final mixture.

Total sugar and reducing sugar contents of samples in each phase were determined using the phenol–sulfuric acid and DNS methods, respectively. The percentage of hydrolysis was calculated as described by Korakli et al.^36^:

$${\text{Digestion }}\;(\% ) = \frac{{{\text{(final}}\;{\text{reducing}}\;{\text{sugar}}\;{\text{content }} - {\text{initial}}\;{\text{reducing}}\;{\text{sugar}}\;{\text{content)}}\;{\text{ppm}}}}{{{\text{(total}}\;{\text{sugar}}\;{\text{content }} - {\text{initial}}\;{\text{reducing}}\;{\text{sugar}}\;{\text{content)}}\;{\text{ppm}}}} \times 100$$

### Statistical analysis

Three independent trials were conducted for each treatment, with mean values and standard deviations of the data calculated. Statistical analyses were carried out using SPSS 11 software. Duncan’s one-way multiple comparisons were performed to determine significant differences (*P* < 0.05).

## Results

### Chemical and carbohydrate composition of RH

**Figure 1:**
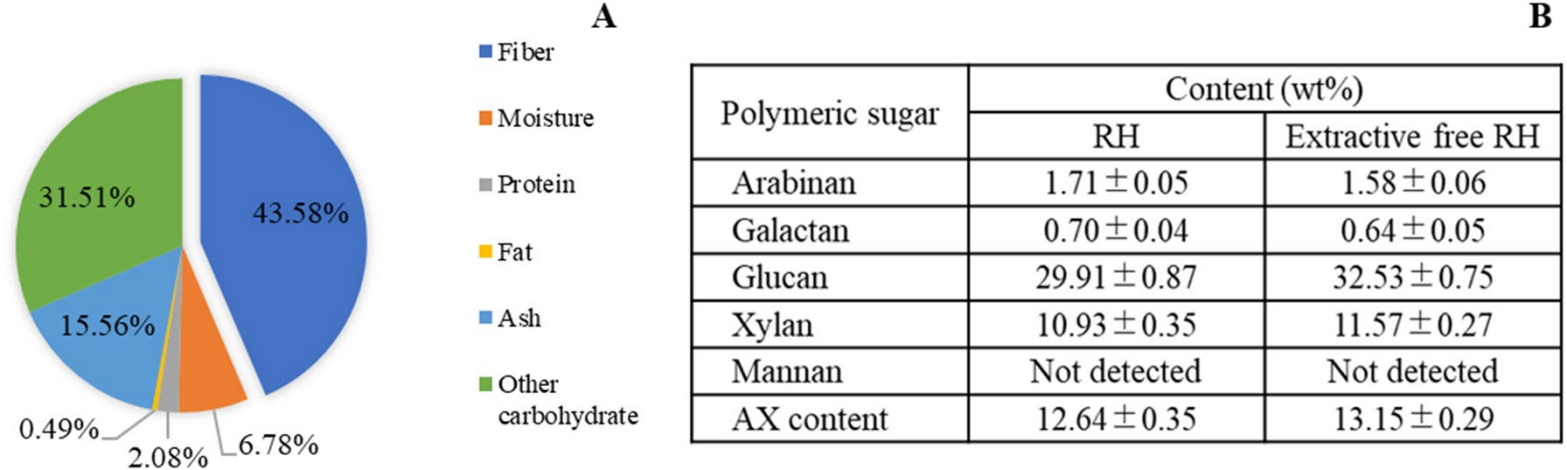
Chemical composition of RH (**A**) and polymeric sugar content of RH and extractive free RH (**B**). Abbreviation: AX, arabinoxylan. AX content = (arabinose content + xylose content) (wt%) × 0.88.

RH contained mainly fiber at 43.58% , followed by other carbohydrate, ash, moisture protein and fat at 31.51, 15.56, 6.78 2.05 and 0.49%, respectively (Fig. 1A). RH has lignocellulosic fibers, which are formed by cellulose, hemicellulose and lignin. RH is also highly siliceous and different from other biomass materials. According to previous reviews, RH ranged from 15 to 20% of ash content. However, values depended on the state of prevention of RH degradation after harvesting^37^. RH is composed of three primary components as cellulose, hemicelluloses and lignin. Di Blasi et al.^38^ demonstrated that rice husk was a good source of lignocellulosic materials, comprising 28.6% hemicellulose, 28.6% cellulose, 24.4% lignin and 18.4% extractive matter. The cellulose and hemicellulose components are both polysaccharides that can be converted to functional sugars. The main hemicellulose compound in RH is xylan. This is made up of substituted arabinoxylan that can be used as a precursor for XOS and AXOS production^39,40^.

Lignocellulosic fiber of RH was divided into polymeric sugar, consisting of 1.71% arabinan, 0.70% galactan, 29.91% glucan and 10.93% xylan. However, extractive-free RH after pretreatment with acetone and ethanol consisted of 1.58% arabinan, 0.64% galactan, 32.53% glucan and 11.57% xylan (Fig. 1B). Glucose is a sugar with six carbon atoms that is found in most plant structures. However, glucose can be absorbed in the upper gastrointestinal tract and enhances the growth of pathogens in human microbiota, exhibiting no prebiotic properties. Interestingly, RH also contains a high amount of xylose as 5 carbon atom sugar that enhances the growth of probiotics, while most pathogens cannot use it as a carbon source^41^. Therefore, oligosaccharides consisting of xylose oligomers in RH are interesting functional ingredients as prebiotic compounds. Hemicellulose is a complex component of the plant cell wall, which is associated with cellulose and lignin in a hetero-matrix form. Hemicellulose forms ether and ester bonds with lignin, while hydrogen bonds form with cellulose^42^. Pretreatment of lignocellulosic materials increases biological conversion yields by disrupting interpolymer linkages and allowing the lignocellulose fractions to separate.

### Effects of microwave treatment on AX extraction

Hemicellulose extraction with an alkaline reagent and subsequent hydrolysis in the presence of acid or enzyme is preferred, due to its high XOS output yield from sugarcane bagasse^43^. Furthermore, AX alkaline extraction can be assisted by microwaves because the energy is uniformly distributed throughout the material as opposed to conventional heating. Process time can thus be reduced, resulting in greater efficiency and homogeneity^20,44^. Microwave treatment causes fragmentation and swelling, leading to lignin and hemicellulose degradation in the biomass and improving the pentose yield^45^. Table 1 shows that AX content slightly decreased after microwave pretreatment at 140–180 °C for 5–15 min with increasing temperature and processing time. The highest AX content of 9.01 g/100 was recorded after 5 min at 140 °C showing no significant difference with 15 min at 180 °C. However, A/X ratio of the AX of 180 °C for 15 min was lower indicating less branches of arabinoxylan that can ease the xylanase enzyme to access the arabinoxylan backbone in hydrolysis process^46^.

**Table 1:** Water-unextractable arabinoxylan content after microwave processing.

Temperature (°C)	Time (min)	Content (wt%)			A/X ratio
		A1	X1	AX content	
140	5	2.08 ± 0.12	8.61 ± 0.54	9.01 ± 0.30^a^	0.24
	10	1.88 ± 0.08	7.79 ± 0.25	8.51 ± 0.24^bc^	0.24
	15	1.81 ± 0.24	8.15 ± 0.20	8.76 ± 0.18^b^	0.22
160	5	1.80 ± 0.16	6.88 ± 0.30	8.31 ± 0.23^cd^	0.26
	10	1.84 ± 0.09	7.60 ± 0.11	7.64 ± 0.07^e^	0.24
	15	1.75 ± 0.14	7.29 ± 0.04	7.96 ± 0.05^cd^	0.24
180	5	1.68 ± 0.20	7.70 ± 0.18	8.25 ± 0.21^cd^	0.22
	10	1.68 ± 0.04	6.89 ± 0.15	7.54 ± 0.10^e^	0.24
	15	1.69 ± 0.15	8.47 ± 0.08	8.94 ± 0.15^a^	0.20

Values with different superscript letters in the same column are significantly different (*P* < 0.05).

Abbreviations: A1, arabinose; X1, xylose; AX; arabinoxylan.

AX content = (arabinose content + xylose content) (wt%) × 0.88.

**Figure 2:**
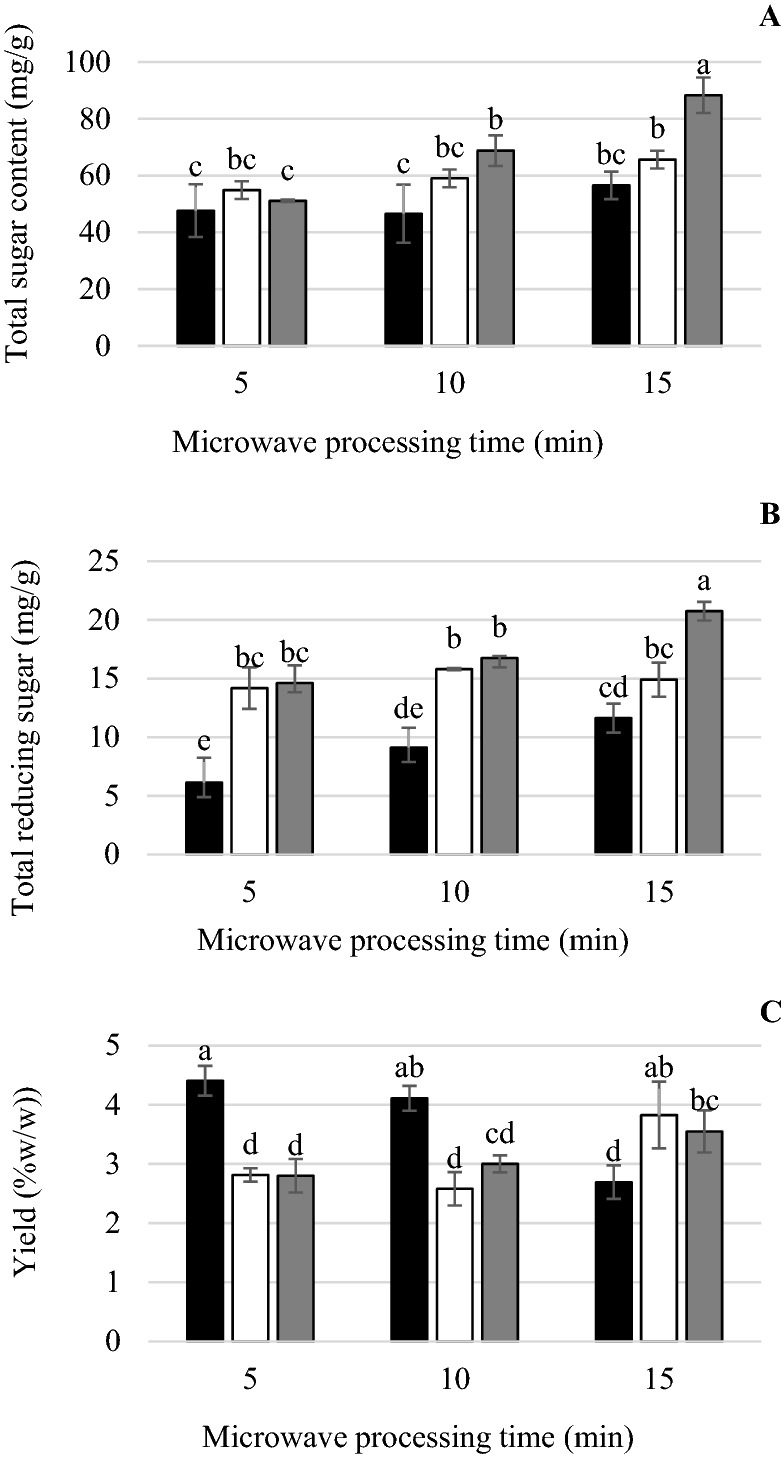
Total reducing sugar content (**A**), total sugar content (**B**) and yield (**C**) by RH (1.5 g in 45 mL of water) after microwave processing at 140 °C (black square), 160 °C (white square) and 180 °C (grey square) for 5–15 min. For each temperature and time of microwave treatment, different superscript letters indicate significant difference (*P* < 0.05).

Figure 2A, B show that reducing sugar and total sugar content increased significantly with increasing temperature during microwave treatment. Microwave treatment at 180 °C for 15 min gave the highest reducing sugar and total sugar contents of 20.75 and 88.3 mg/g, respectively. The preliminary experiment, alkali extraction of RH without microwave treatment, provided reducing sugar and total sugar contents of 0.9 and 48.4 mg/g (data not shown). Operating parameters that affected microwave conversion efficiency of sugar-biomass assisted pretreatment were discussed by Ethaib et al.^47^. They reported that biomass load, power level of the microwave and time of irradiation all affected the efficiency of microwave pretreatment to convert biomass into sugar. Microwave-assisted pretreatment of lignocellulosic biomass immersed in alkaline glycerol showed increased enzyme hydrolysis of corn straw and rice husk to sugar production^48^. The AX concentration in spent grains from the brewing process was maximized under pretreatment conditions of microwave heating at 172 °C and 0.38 M sodium hydroxide^49^. Microwave processing at 140 °C for 5 min provided the highest AX content but the A/X ratio was high, as shown in Table 1. Lafond et al.^46^ reported that enzymes were less sensitive to an increase in the A/X ratio at high A/X ratio. The effect of A/X ratio on enzymatic hydrolysis was determined and results revealed that extracted AX with a lower A/X ratio had higher reducing sugar content (data not shown). Therefore, the optimal condition for hemicellulose extraction was 180 °C for 15 min, providing 3.5% AX yield of RH, as shown in Fig. 2C. For XOS and AXOS production, extracted RH-AX was further hydrolysed by commercial xylanase.

**Figure 3:**
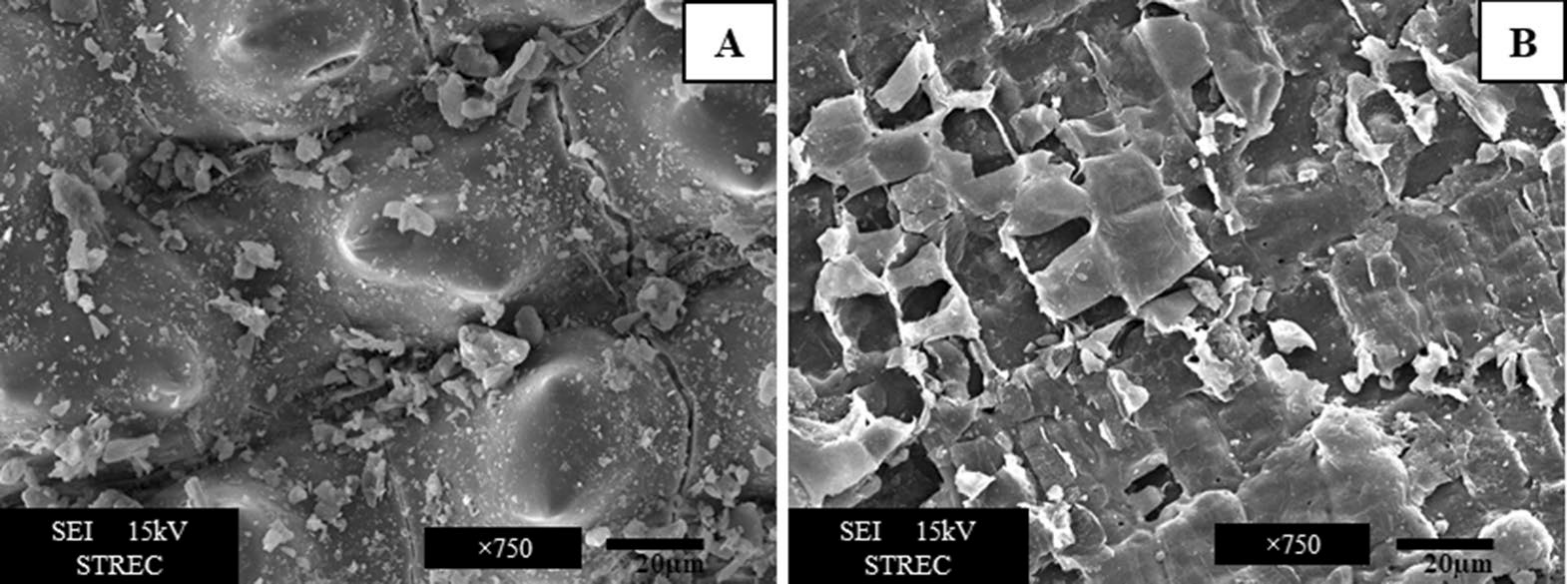
Scanning electron microscopy (SEM) analysis of untreated RH (**A**) and the pretreated RH (**B**) with microwave at 180 °C for 15 min.

The preliminary study compared the activity of endoxylanases in hydrolysis of untreated and microwave-treated RH. Results showed that in microwave-treated RH, the reducing sugar content was more than 2 folds higher compared with the untreated sample (data not shown). SEM was used to investigate the surface morphology of RH before and after microwave pretreatment. SEM images of untreated and treated RH showed remarkable changes in the surface structure. As shown in Fig. 3A, the untreated RH surface had a packed structure with the composition of the plant cell wall and some convex structures. According to the baseline data of raw RH, the outer surface of the raw RH consisted mainly of carbon, oxygen, nitrogen and silica. The complex lignocellulosic structure of RH has very low lignocellulosic-degrading enzyme permeability. Microwave pretreatment was expected to degrade the cell wall sufficiently and increase the surface area of the RH structure to allow direct contact with the degrading enzyme. Figure 3B shows that the surface morphology of the treated RH varied remarkably. Thermal treatment induced fragmentation and swelling, leading to lignin and hemicellulose degradation in the biomass^50^.

Alkaline pretreatment enhanced enzymatic hydrolysis by selectively removing lignin without damaging the carbohydrates and increasing porosity and surface area^51^. Binod et al.^52^ reported that the crystallinity index of native sugarcane bagasse was lower than other pretreated samples, according to the X-ray diffraction profiles of native and microwave-pretreated samples. Crystalline size of native sugarcane bagasse was greater than the pretreated sugarcane bagasse due to degradation of the linkages between lignin and hemicellulose and subsequent removal of lignin, which increased the surface area. Furthermore, non-thermal effects of exposure degraded the lignocellulose structure, while other physical parameters caused fluctuation and vibration of the charged particles and tissues^53^. Wang and Lu^54^ reported that the effect of microwave treatment on XOS production from wheat bran with xylanase was characterized by rapid increase in the reducing sugar by almost 3–4 folds, compared with untreated wheat bran. Microwave alkaline extraction of AX produced various lignocellulosic residues. Roos et al.^55^ reported that microwave treatment considerably enhanced the xylose ratio of barley husks, while Jiang et al.^56^ reported that ultrasonic-microwaves synergetically increased arabinoxylan content in corn bran more than the conventional method.

### Effects of commercial xylanases on XOS and AXOS production

**Figure 4:**
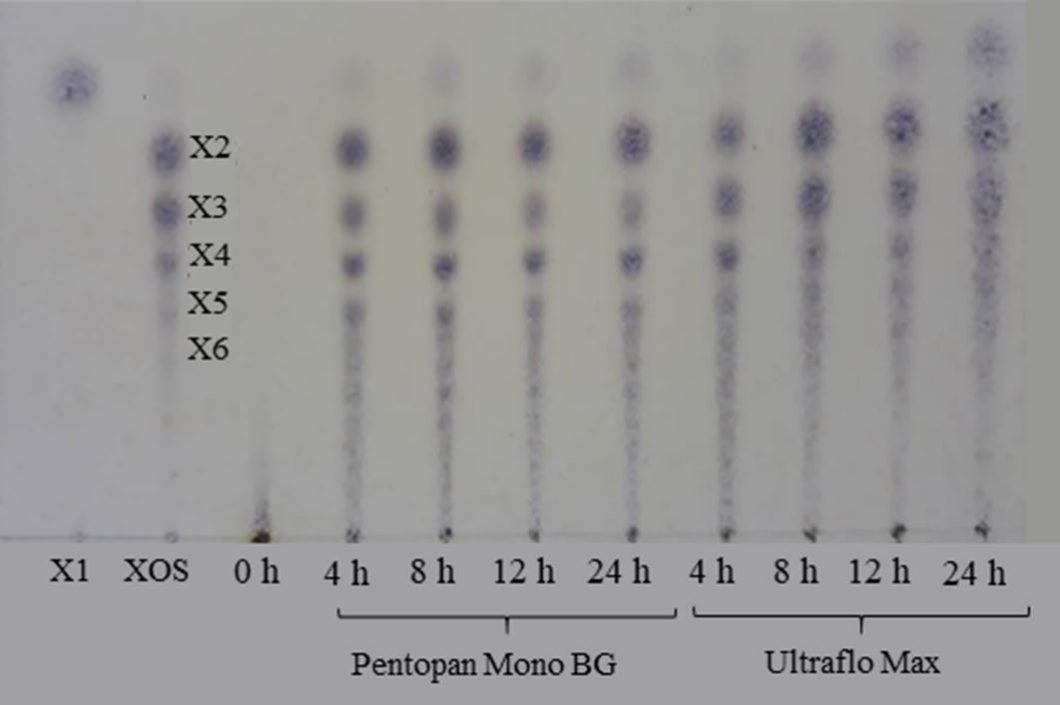
TLC chromatograms of XOS obtained from RH-AX after hydrolysis with different commercial xylanases at 50 U/g, 50 °C, pH 6.0 for 0–24 h. Mixtures X1–X6 were used as standards.

Oligosaccharide profiles were observed by TLC during 0–24 h of incubation, compared to mixtures of X1-X6 as standards (Fig. 4). The TLC did not exhibit a sugar band before RH-AX was incubated with xylanases, indicating that it could be a long-chain carbohydrate. Incubations using Pentopan Mono BG and Ultraflo Max hydrolysed long-chain carbohydrate into short-chain oligosaccharides. The TLC results confirmed that both enzymes gave similar sugar patterns, including X1, X2, X3 X4 and X5. However, Ultraflo Max hydrolysed the substrate to a degree of polymerisation (DP) and produced predominantly more monosaccharides than Pentopan Mono BG. XOS produced by Pentopan Mono BG provided various kinds of oligosaccharides from X2 to X5.

**Figure 5:**
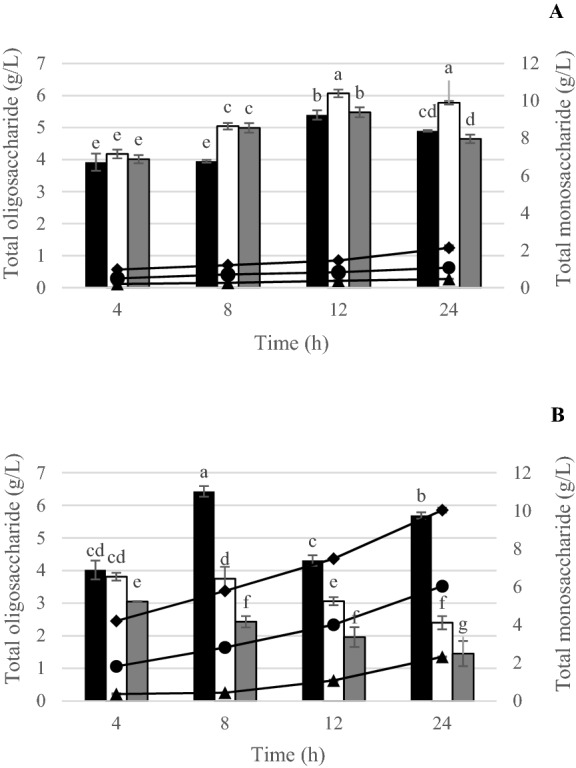
Bars showing total oligosaccharide contents when treating RH-AX with Pentopan Mono BG (**A**) and Ultraflo Max (**B**) at 50 (black square), 150 (white square) and 300 (grey square) U/g substrate of enzyme concentration. Solid lines with markers show total monosaccharide content hydrolysate when treating RH-AX with the commercial xylanases at 50 (black filled triangle), 150 (black filled circle) and 300 (black filled diamond) U/g substrate of enzyme concentration at 50 °C, pH 6.0 for 0–24 h. Different superscripts for each enzyme concentration and time of enzyme hydrolysis represent significant differences (*P* ≤ 0.05).

Furthermore, the oligosaccharide hydrolysates of RH-AX produced by both commercial xylanases were analyzed by HPAEC for sugar patterns. Results demonstrated that Pentopan Mono BG produced oligosaccharide hydrolysate with the highest total oligosaccharide content at 150 U/g of enzyme concentration, after 12 and 24 h of incubation (Fig. 5A), while the Ultraflo Max produced oligosaccharide hydrolysate with the highest total oligosaccharide content at 50 U/g of enzyme concentration after 8 h of incubation (Fig. 5B). The presence of exo-xylanases in the commercial enzyme gave increased concentration and incubation time with increased total monosaccharide content. Increased concentration allowed undesirable enzyme activity to hydrolyse the short-chain oligomer into monosaccharide, resulting in a significant decrease in XOS production. Furthermore, the longer an enzyme was incubated with its substrate, the greater the hydrolysis activity and amount of smallest products formed.

**Figure 6:**
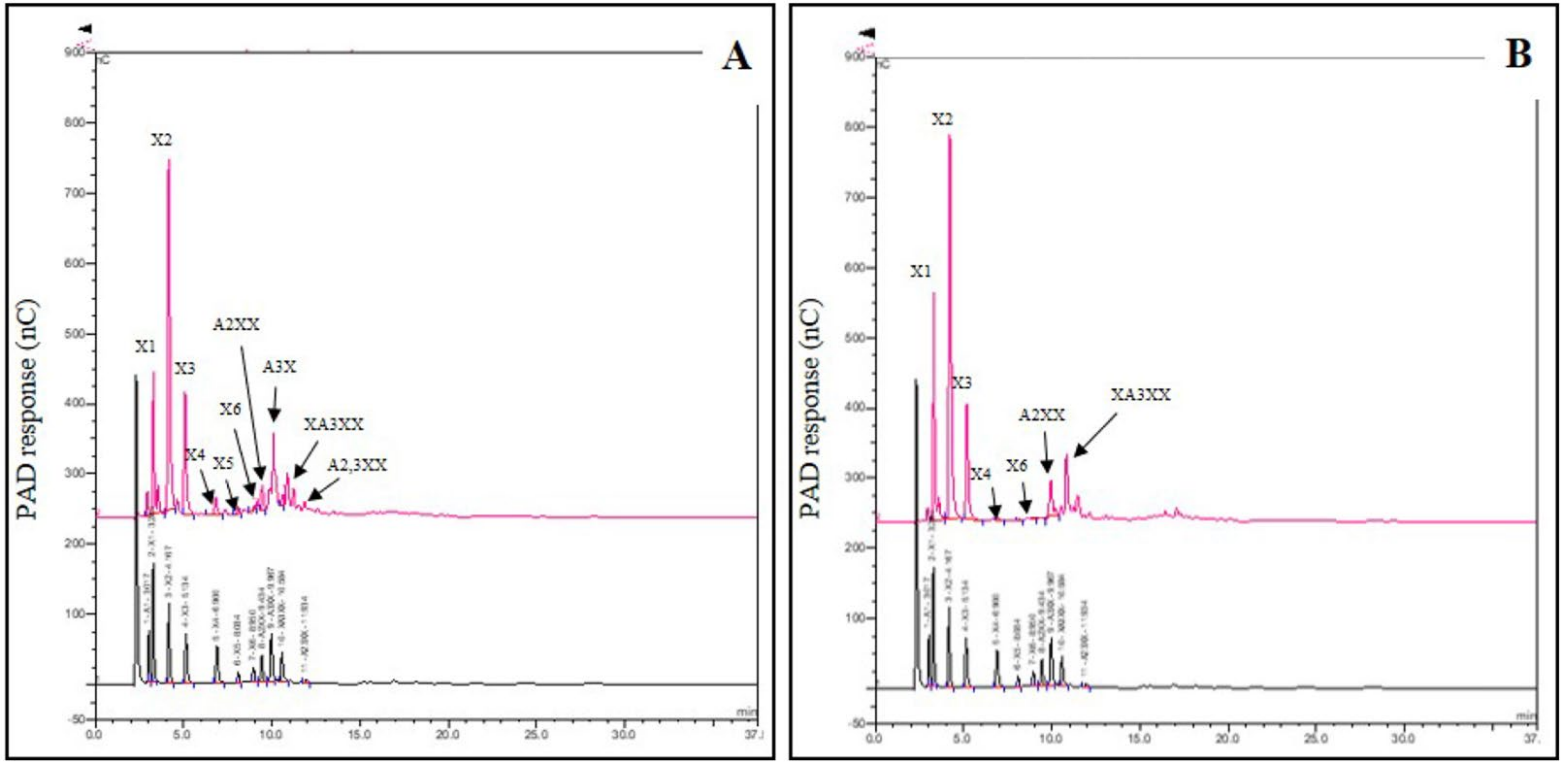
HPAEC chromatograms of oligosaccharides from RH-AX treated with Pentopan Mono BG at 150 U, 50 °C for 12 h (**A**) and Ultraflo Max at 50 U, 50 °C for 8 h (**B**).

The HPAEC chromatograms showed that both commercial enzymes provided X2 and X3 as the main oligosaccharide found in the hydrolysates of RH-AX, followed by X4, X5 and X6 as minor oligosaccharide. Interestingly, AXOS was found in both hydrolysates (Fig. 6A, B). Quantitative analyses of XOS and AXOS produced by both commercial xylanases are shown in Supplementary Tables S1 and S2. The oligosaccharide produced by Pentopan Mono BG exhibited A2XX, A3X, XA3XX and A2,3XX as 55.96, 784.68, 171.20 and 687.55 mg/L, respectively while the oligosaccharide produced by Ultraflo Max exhibited A2XX and XA3XX as 670.72 and 590.46 mg/L, respectively. In this study, oligosaccharide produced by Pentopan Mono BG was then used to evaluate the prebiotic properties because it contained less monosaccharide and various kinds of oligosaccharides.

In the carbohydrate-active enzyme (CAZY) database, xylanases are classified based on primary structure comparisons of the catalytic domains and grouped in families of related sequences. Members of the same family have similar protein folds, the same catalytic mechanism and anomeric carbon retention or inversion. Enzymes from glycoside hydrolase families 5, 7 and 8 contain a catalytic domain with endo-1,4 xylanase activity but research has primarily focused on xylanases from glycoside hydrolase families 10 and 11^57^. Pentopan Mono BG and Ultraflo Max belong to the glycoside hydrolase families 10 and 11, showing some differences in sugar profiles^58^. Family 11 xylanases hydrolyse only xylan and generally exhibit a preference for internal xylan bonds and unsubstituted xylan chains. They act primarily on the xylose unit at the center of the oligosaccharide. Conversely, family 10 xylanases tend to show preference for groups at the end of the xylan bonds, especially favoring the reducing end. They have low substratum specificity and can degrade xylan backbones with several replacements by hydrolysing at the side branch location^59^. Pentopan mono BG produced XOS from *Phoenix dactylifera* L. seed with higher amounts of X3 than X2^60^. Falk et al.^61^ reported that GH10 xylanases produced a higher amount of short-chain XOS from rye bran than GH11. According to the datasheet of the enzymes, GH10 prefers to hydrolyse xylan into short-chain XOS, while GH11 prefers to hydrolyse into long-chain XOS.

### Growth promotion of lactic acid bacteria by RH-XOS

A prebiotic is a selectively fermented ingredient that allows for specific changes in the composition and/or activity of the gastrointestinal microbiota, which confers benefits. It is necessary to establish clear criteria for classifying a food ingredient as a prebiotic^62^. XOS and AXOS possess promising functional properties as they can be specifically fermented by intestinal commensals such as bifidobacteria and lactobacilli^63^. XOS has high prebiotic potential and can be incorporated into a wide range of food products. Seven strains of lactic acid bacteria were used to evaluate the ability of RH-XOS to promote growth compared to xylose and commercial prebiotics, including XOS95P, RMD and inulin. Table 2 shows the utilization of RH-XOS and commercial prebiotics by lactic acid bacteria strains. XOS95P and RMD promoted the growth of all the strains, whereas RH-XOS did not promote *L. Lactis*. Only *L. brevis* grew well on xylose. A previous study also found that *L. brevis* grew better on xylose than on a glucose and xylose mixture^64^. Inulin and RH-XOS promoted six strains except *L. brevis*. Kariyawasam et al.^65^ revealed that inulin partially promoted *L. brevis* strains, while *L. brevis* displayed high growth and consumption in XOS^66^. The results of this study also demonstrated that the ability of probiotic bacteria to utilize prebiotics varies, even within the same species. Iliev et al.^67^ reported that XOS with different DPs stimulated the growth of some heterofermentative *Lactobacillus* strains. Approximately 30 strains were identified and screened for XOS utilization. Results showed that the three enzymes β-xylosidase, exo-oligoxylanase and α-L-arabinofuranosidase could alter the end-products and morphology of *Lactobacillus* strain growth by XOS.

**Table 2:** Carbohydrate utilization by lactic acid bacteria.

Microorganism	Carbohydrate sources				
	Xylose	XOS95P	RMD	Inulin	RH-XOS
*Lactobacillus johnsonii*	−	+	+	+	+
*Lactobacillus plantarum*	−	+	+	+	+
*Lactobacillus reuteri*	−	+	+	+	+
*Lactobacillus bulgaricus*	−	+	+	+	+
*Lactobacillus sakei*	−	+	+	+	+
*Lactococcus lactic*	−	+	+	+	−
*Lactobacillus brevis*	+	+	+	−	+

+ : Carbohydrates can promote growth of lactic acid bacteria.

− : Carbohydrate cannot promote growth of lactic acid bacteria.

*XOS95P* 95% commercial XOS, *RMD* resistant maltodextrin, *RH-XOS* xylo-oligosaccharide obtained from rice husk.

### In vitro* simulation of human digestibility of RH-XOS*

**Figure 7:**
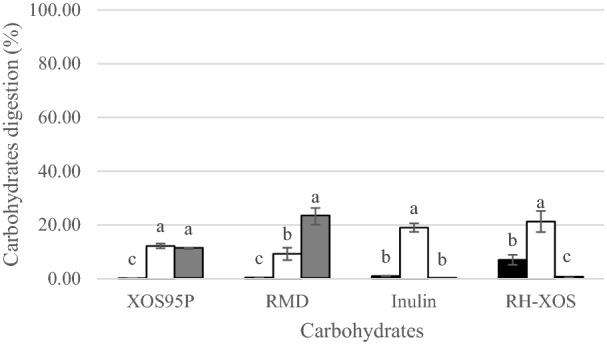
Percentage of digested carbohydrate in in vitro simulated digestion in oral phase (black square), gastric phase (white square) and intestinal phase (grey square). For each simulated digestion phase, different superscript letters show significant difference (*P* < 0.05).

Human-simulated digestion typically includes the oral, gastric and small intestinal phases. These phases were performed to study the digestion resistance property of RH-XOS compared with commercial prebiotics. Figure 6 shows that the carbohydrate used in this study was significantly hydrolysed in the gastric phase and small intestinal phase. Commercial XOS and RMD were not digested in the oral phase because commercial XOS mostly contain short-chain oligomers, which are less than DP 4. Alpha-amylase is a glycoside hydrolase family 13, which catalyses the hydrolysis of (1–4)-α-d-glucosidic linkages in polysaccharides. Substrates are not digested by α-amylase, such as α-limit dextrin, and small linear oligomers, along with larger α-glucans^68^. By contrast, inulin and RH-XOS were digested in small amounts in the oral phase at 1.01% and 7.08%, respectively. In a previous study, commercial XOS (DP 2–4) and inulin did not degrade when incubated with salivary amylase enzyme but they may have an impact on digestion, which is defined as the hydrolytic and other processes that occur in the stomach and small intestine^69,70^. This result could be due to an impure composition hydrolysed by salivary α-amylase, which catalyzed the hydrolysis of α-glycosidic bonds of polysaccharide starch^71^. In the gastric phase, the percentage of digestion increased in all samples, especially inulin and RH-XOS at 19.05% and 21.30%, respectively as the stomach plays an enhanced role in hydrolysis by hydrochloric acid^68^. Commercial XOS was digested in the gastric phase and small intestinal phase at the same digestion level of 12.21% and 11.23%, respectively. Inulin and RH-XOS were less digested in the intestinal phase, while RMD was more digested in the intestinal phase, (23.24%) (Fig. 7). Exo-glucosidases that act on the non-reducing end of glucose oligomers and catalyse not only the hydrolysis of α-1,4-glycosidic bonds, but also to a lesser extent α-1,6 glycosidic bonds, ensuring further degradation of nonlinear oligosaccharides^68^. This study found that the obtained RH-XOS was more than 70% resistant to human simulated digestion, similar to commercial prebiotics. According to previous research, XOS are resistant to hydrolysis by enzymes and/or the low pH found in human saliva, gastric, and pancreatic juices. XOS are not absorbed during transit through the small intestine^68^. Eventually the XOS are able to reach the colon and serve as fermentable substrates for certain members of the resident.

## Conclusion

Results showed that microwave pretreatment is a promising method for lignocellulosic-degradation of RH. Commercial xylanases, Pentopan Mono BG, and Ultraflo Max were able to hydrolyse RH-AX into XOS and AXOS. Pentopan Mono BG provided fewer monosaccharides and various kinds of oligosaccharides compared to Ultraflo Max. RH-XOS prepared by Pentopan Mono BG promoted growth of six of the seven lactic acid bacterial species, and was resistant to human simulated digestion by more than 70%, indicating high prebiotic potential.

## Supplementary Information

Supplementary Tables.

## References

[1] Worasuwannarak N, Sonobe T, Tanthapanichakoon W (2007). Pyrolysis behaviors of rice straw, rice husk, and corncob by TG-MS technique. J. Anal. Appl. Pyrolysis.

[2] Hu B (2021). On the mechanism of xylan pyrolysis by combined experimental and computational approaches. Proc. Combust. Inst..

[3] Dornez E, Gebruers K, Delcour JA, Courtin CM (2009). Grain-associated xylanases: Occurrence, variability, and implications for cereal processing. Trends Food Sci. Technol..

[4] Abouloifa H (2020). The prebiotics (Fructo-oligosaccharides and Xylo-oligosaccharides) modulate the probiotic properties of Lactiplantibacillus and Levilactobacillus strains isolated from traditional fermented olive. World J. Microbiol. Biotechnol..

[5] Singh RD, Banerjee J, Arora A (2015). Prebiotic potential of oligosaccharides: A focus on xylan derived oligosaccharides. Bioact. Carbohydr. Diet. Fiber.

[6] Terrasan CR, de Morais Junior WG, Contesini FJ (2019). Enzyme Immobilization for Oligosaccharide Production.

[7] Ávila PF, Martins M, de Almeida Costa FA, Goldbeck R (2020). Xylooligosaccharides production by commercial enzyme mixture from agricultural wastes and their prebiotic and antioxidant potential. Bioact. Carbohydr. Diet Fiber..

[8] Stahl, B., Zens, Y. & Boehm, G. Prebiotics with special emphasis on fructo-, galacto-, galacturono-, and xylooligosaccharides (2007).

[9] Mathew S, Aronsson A, Karlsson EN, Adlercreutz P (2018). Xylo-and arabinoxylooligosaccharides from wheat bran by endoxylanases, utilisation by probiotic bacteria, and structural studies of the enzymes. Appl. Microbiol. Biotechnol..

[10] Juturu V, Wu JC (2014). Microbial exo-xylanases: A mini review. Int. J. Appl. Biotechnol. Biochem..

[11] Akpinar O, Erdogan K, Bostanci S (2009). Enzymatic production of xylooligosaccharide from selected agricultural wastes. Food Bioprod. Process..

[12] Biely P, Vršanská M, Tenkanen M, Kluepfel D (1997). Endo-β-1, 4-xylanase families: Differences in catalytic properties. J. Biotechnol..

[13] Dodd D, Cann IK (2009). Enzymatic deconstruction of xylan for biofuel production. GCB Bioenergy.

[14] Lam ND, Nagasawa N, Kume T (2000). Effect of radiation and fungal treatment on lignocelluloses and their biological activity. Radiat. Phys. Chem. Oxf. Engl..

[15] Aguilar-Reynosa A (2017). Comparison of microwave and conduction-convection heating autohydrolysis pretreatment for bioethanol production. Bioresour. Technol..

[16] Hoang AT (2021). Insight into the recent advances of microwave pretreatment technologies for the conversion of lignocellulosic biomass into sustainable biofuel. Chemosphere.

[17] Gabhane J, William SP, Vaidya AN, Mahapatra K, Chakrabarti T (2011). Influence of heating source on the efficacy of lignocellulosic pretreatment–a cellulosic ethanol perspective. Biomass Bioenergy.

[18] Gissibl A (2018). Microwave pretreatment of paramylon enhances the enzymatic production of soluble β-1, 3-glucans with immunostimulatory activity. Carbohydr. Polym..

[19] Palm M, Zacchi G (2003). Extraction of hemicellulosic oligosaccharides from spruce using microwave oven or steam treatment. Biomacromol.

[20] Coelho E, Rocha MAM, Saraiva JA, Coimbra MA (2014). Microwave superheated water and dilute alkali extraction of brewers’ spent grain arabinoxylans and arabinoxylo-oligosaccharides. Carbohydr. Polym..

[21] Kundu P, Kumar S, Ahluwalia V, Kansal SK, Elumalai S (2018). Extraction of arabinoxylan from corncob through modified alkaline method to improve xylooligosaccharides synthesis. Bioresour. Technol. Rep..

[22] Gibson GR (2017). Expert consensus document: The International Scientific Association for Probiotics and Prebiotics (ISAPP) consensus statement on the definition and scope of prebiotics. Nat. Rev. Gastroenterol. Hepatol..

[23] Swennen K, Courtin CM, Delcour JA (2006). Non-digestible oligosaccharides with prebiotic properties. Crit. Rev. Food. Sci. Nutr..

[24] AOAC, B. A. M. Association of official analytical chemists. Official methods of analysis, Vol. 12 (2000).

[25] Jaichakan P, Thi HND, Nakphaichit M, Klangphetch W (2019). The Effect of alkali pretreatment and acid debranching on rice husk, rice straw and defatted rice bran for xylobiose production by commercial xylanases. J. Sci. Technol..

[26] McCleary BV (2015). Hydrolysis of wheat flour arabinoxylan, acid-debranched wheat flour arabinoxylan and arabino-xylo-oligosaccharides by β-xylanase, α-L-arabinofuranosidase and β-xylosidase. Carbohydr. Res..

[27] Sluiter A (2008). Determination of structural carbohydrates and lignin in biomass. Lab. Anal. Proc..

[28] Dubois M, Gilles KA, Hamilton JK, Rebers PT, Smith F (1956). Colorimetric method for determination of sugars and related substances. Anal. Chem..

[29] Miller GL (1959). Use of dinitrosalicylic acid reagent for determination of reducing Miller sugar. Anal. Chem..

[30] Jaichakan P, Nhung DTH, Nakphaichit M, Klangpetch W (2019). Intensification of cellulolytic hydrolysis of rice husk, rice straw, and defatted rice bran by sodium hydroxide pretreatment. FAB J..

[31] Prommadee P, Garnjanagoonchorn W, de Lange K, Nitisinprasert S (2012). Characterization of Lactobacillus johnsonii KUNN19-2 and Pediococcus pentosaceus KUNNE6-1 isolated from thai-style fermented pork (Nham) for their probiotic properties in the gastrointestinal tract and immunomodulation. Agric. Nat. Resour..

[32] Sobanbua S (2020). Cloning and expression of the antimicrobial peptide from *Lactobacillus reuteri* KUB-AC5 and its characterization. Technology.

[33] Plupjeen SN, Chawjiraphan W, Charoensiddhi S, Nitisinprasert S, Nakphaichit M (2020). Lactococcus lactis KA-FF 1–4 reduces vancomycin-resistant enterococci and impacts the human gut microbiome. 3 Biotech.

[34] Nakphaichit M (2011). The effect of including *Lactobacillus reuteri* KUB-AC5 during post-hatch feeding on the growth and ileum microbiota of broiler chickens. Poult. Sci..

[35] Minekus M (2014). A standardised static in vitro digestion method suitable for food–an international consensus. Food Func..

[36] Korakli M, Gänzle MG, Vogel RF (2002). Metabolism by bifidobacteria and lactic acid bacteria of polysaccharides from wheat and rye, and exopolysaccharides produced by Lactobacillus sanfranciscensis. J. Appl. Microbiol..

[37] Ismail MS, Waliuddin AM (1996). Effect of rice husk ash on high strength concrete. Constr. Build. Mater..

[38] Di Blasi C, Signorelli G, Di Russo C, Rea G (1999). Product distribution from pyrolysis of wood and agricultural residues. Ind. Eng. Chem. Res..

[39] Garrote GDHP, Dominguez H, Parajo JC (1999). Hydrothermal processing of lignocellulosic materials. Holz als roh-und werkstoff.

[40] Vegas R, Alonso JL, Domínguez H, Parajó JC (2004). Processing of rice husk autohydrolysis liquors for obtaining food ingredients. J. Agric. Food Chem..

[41] Desai MS (2016). A dietary fiber-deprived gut microbiota degrades the colonic mucus barrier and enhances pathogen susceptibility. Cell.

[42] Harmsen, P. F., Huijgen, W., Bermudez, L. & Bakker, R. Literature review of physical and chemical pretreatment processes for lignocellulosic biomass (No. 1184). Wageningen UR-Food & Biobased Research (2010).

[43] Brienzo M, Carvalho W, Milagres AM (2010). Xylooligosaccharides production from alkali-pretreated sugarcane bagasse using xylanases from Thermoascus aurantiacus. Appl. Biochem. Biotechnol..

[44] Bastos, R., Coelho, E. & Coimbra, M. A. Arabinoxylans from cereal by-products: Insights into structural features, recovery, and applications. In: *Sustainable Recovery and Reutilization of Cereal Processing By-Products* 227–251 (Woodhead Publishing, 2018).

[45] Chen WH, Tu YJ, Sheen HK (2011). Disruption of sugarcane bagasse lignocellulosic structure by means of dilute sulfuric acid pretreatment with microwave-assisted heating. Appl. Energy.

[46] Lafond M, Guais O, Maestracci M, Bonnin E, Giardina T (2014). Four GH11 xylanases from the xylanolytic fungus Talaromyces versatilis act differently on (arabino) xylans. Appl. Microbiol. Biotechnol..

[47] Ethaib S, Omar R, Kamal SM, Biak DA (2015). Microwave-assisted pretreatment of lignocellulosic biomass: A review. J. Eng. Sci. Technol..

[48] Diaz AB (2015). Evaluation of microwave-assisted pretreatment of lignocellulosic biomass immersed in alkaline glycerol for fermentable sugars production. Bioresour. Technol..

[49] López-Linares JC, Lucas S, García-Cubero MT, Jiménez JJ, Coca M (2020). A biorefinery based on brewers spent grains: Arabinoxylans recovery by microwave assisted pretreatment integrated with butanol production. Ind. Crops Prod..

[50] Puligundla P, Oh SE, Mok C (2016). Microwave-assisted pretreatment technologies for the conversion of lignocellulosic biomass to sugars and ethanol: A review. Carbon Lett..

[51] Kim JS, Lee YY, Kim TH (2016). A review on alkaline pretreatment technology for bioconversion of lignocellulosic biomass. Bioresour. Technol..

[52] Binod P (2012). Short duration microwave assisted pretreatment enhances the enzymatic saccharification and fermentable sugar yield from sugarcane bagasse. Renew. Energy.

[53] Belyaev I (2005). Non-thermal biological effects of microwaves. Microw. Rev..

[54] Wang TH, Lu S (2013). Production of xylooligosaccharide from wheat bran by microwave assisted enzymatic hydrolysis. Food Chem..

[55] Roos AA, Persson T, Krawczyk H, Zacchi G, Stålbrand H (2009). Extraction of water-soluble hemicelluloses from barley husks. Bioresour. Technol..

[56] Jiang Y (2019). Optimization of ultrasonic-microwave assisted alkali extraction of arabinoxylan from the corn bran using response surface methodology. Int. J. Biol. Macromol..

[57] Collins T, Gerday C, Feller G (2005). Xylanases, xylanase families and extremophilic xylanases. FEMS Microbiol. Rev..

[58] Cheng YS (2014). Structural analysis of a glycoside hydrolase family 11 xylanase from Neocallimastix patriciarum: Insights into the molecular basis of a thermophilic enzyme. J. Biol. Chem..

[59] Morgan NK, Wallace A, Bedford MR, Choct M (2017). Efficiency of xylanases from families 10 and 11 in production of xylo-oligosaccharides from wheat arabinoxylans. Carbohydr. Polym..

[60] Ataei D, Hamidi-Esfahani Z, Ahmadi-Gavlighi H (2020). Enzymatic production of xylooligosaccharide from date (Phoenix dactylifera L.) seed. Food Sci. Nutr..

[61] Falk P (2014). Production of arabinoxylan-oligosaccharide mixtures of varying composition from rye bran by a combination of process conditions and type of xylanase. Bioresour. Technol..

[62] Roberfroid MB (2008). Prebiotics: Concept, Definition, Criteria, Methodologies, and Products.

[63] Neyrinck AM (2012). Wheat-derived arabinoxylan oligosaccharides with prebiotic effect increase satietogenic gut peptides and reduce metabolic endotoxemia in diet-induced obese mice. Nutr. Diabetes.

[64] Kim JH, Shoemaker SP, Mills DA (2009). Relaxed control of sugar utilization in *Lactobacillus brevis*. Microbiology.

[65] Kariyawasam KMGMM, Lee NK, Paik HD (2021). Synbiotic yoghurt supplemented with novel probiotic *Lactobacillus brevis* KU200019 and fructooligosaccharides. Food Biosci..

[66] Moura P (2007). In vitro fermentation of xylo-oligosaccharides from corn cobs autohydrolysis by Bifidobacterium and Lactobacillus strains. LWT.

[67] Iliev I, Vasileva T, Bivolarski V, Momchilova A, Ivanova I (2020). Metabolic profiling of xylooligosaccharides by lactobacilli. Polymers.

[68] Boron WF, Boulpaep EL (2012). Medical Physiology, 2e Updated Edition E-Book: With Student Consult Online Access.

[69] de Figueiredo FC, de Barros Ranke FF, de Oliva-Neto P (2020). Evaluation of xylooligosaccharides and fructooligosaccharides on digestive enzymes hydrolysis and as a nutrient for different probiotics and Salmonella typhimurium. Lwt.

[70] Roberfroid M (1993). Dietary fiber, inulin, and oligofructose: A review comparing their physiological effects. Crit. Rev. Food Sci. Nutr..

[71] Janeček Š, Svensson B, MacGregor EA (2014). α-Amylase: An enzyme specificity found in various families of glycoside hydrolases. Cell. Mol. Life Sci..

