# Publisher Correction: Microwave-assisted enzymatic hydrolysis to produce xylooligosaccharides from rice husk alkali-soluble arabinoxylan

**DOI:** 10.1038/s41598-022-05741-7

**Published:** 2022-02-01

**Authors:** Wannaporn Klangpetch, Alisa Pattarapisitporn, Suphat Phongthai, Niramon Utama-ang, Thunnop Laokuldilok, Pipat Tangjaidee, Tri Indrarini Wirjantoro, Pannapapol Jaichakan

**Affiliations:** 1 Faculty of Agro-Industry, Chiang Mai University, Chiang Mai, 50100 Thailand; 2 Cluster of High Value Products from Thai Rice and Plants for Health, Chiang Mai University, Chiang Mai, 50100 Thailand; 3 Cluster of Innovative Food and Agro-Industry, Chiang Mai University, Chiang Mai, 50100 Thailand; 4 Graduate School of Agriculture, Saga University, Honjo, Saga 840-8502 Japan; 5 Research Center for Development of Local Lanna Rice and Rice Products, Chiang Mai University, Chiang Mai, 50200 Thailand; 6 Department of Agro-Industry, Faculty of Agriculture Natural Resources and Environment, Naresuan University, Phitsanulok, 65000 Thailand

Correction to: *Scientific Reports* https://doi.org/10.1038/s41598-021-03360-2, published online 07 January 2022

The original version of this Article omitted an affiliation for Wannaporn Klangpetch, Niramon Utama-ang, Thunnop Laokuldilok, Pipat Tangjaidee and Tri Indrarini Wirjantoro. This affiliation is listed below.

Cluster of Innovative Food and Agro-Industry, Chiang Mai University, Chiang Mai 50100, Thailand

The original Article has been corrected.

