# Supplementary material for: Microwave-assisted enzymatic hydrolysis to produce xylooligosaccharides from rice husk alkali-soluble arabinoxylan
Source: Sci Rep. 2022 Jan 7;12:11. doi: 10.1038/s41598-021-03360-2 (PMC8741828; doi:10.1038/s41598-021-03360-2)
Supplement: Supplementary file 1 — Supplementary Tables. [file 41598_2021_3360_MOESM1_ESM.docx]

**Supplementary Table S1**

Quantitative analysis of XOS and AXOS produced by Pentopan Mono BG

| **Enzyme Conc. (U/g substrate)** | **Time (h)** | **Sugar content (ppm)** | | | | | | | | | | |
| --- | --- | --- | --- | --- | --- | --- | --- | --- | --- | --- | --- | --- |
|  |  | **A1** | **X1** | **X2** | **X3** | **X4** | **X5** | **X6** | **A2XX** | **A3X** | **XA3XX** | **A2,3XX** |
| **50** | **4** | ND | 198.95±32.82 | 1543.05±57.46 | 1274.41±96.38 | 257.14±37.46 | ND | ND | ND | 374.90±16.39 | ND | 468.92±60.02 |
|  | **8** | ND | 267.87±3.50 | 1834.02±32.15 | 1220.93±6.09 | 93.99±1.15 | 31.68±3.92 | 135.63±16.12 | 11.74±0.65 | 346.86±8.25 | ND | 271.47±6.40 |
|  | **12** | ND | 374.44±43.30 | 1978.72±66.67 | 1192.95±73.97 | 108.82±56.58 | 148.80±25.86 | 203.76±47.11 | 21.98±13.10 | 686.53±138.48 | 192.09±2.61 | 860.69±71.88 |
|  | **24** | ND | 469.83±14.59 | 2337.25±185.02 | 1391.03±85.44 | 103.28±28.18 | 84.23±16.45 | 180.42±6.03 | 19.27±9.55 | 509.99±58.96 | 250.85±78.55 | 20.17±4.95 |
| **150** | **4** | ND | 302.55±106.29 | 1974.43±65.63 | 1116.64±120.60 | 199.05±29.55 | ND | ND | ND | 452.34±14.67 | ND | 432.29±5.83 |
|  | **8** | ND | 438.06±30.38 | 2073.43±44.37 | 974.84±73.86 | 123.82±5.42 | 127.39±12.95 | 217.84±45.03 | 17.77±13.62 | 570.59±46.07 | 149.31±22.04 | 788.76±38.64 |
|  | **12** | ND | 458.60±59.77 | 2426.49±106.87 | 1411.23±120.72 | 144.08±28.81 | 139.13±20.59 | 247.60±21.43 | 55.96±20.76 | 784.68±55.41 | 171.20±11.41 | 687.55±88.87 |
|  | **24** | ND | 603.64±56.14 | 2658.52±102.18 | 1234.46±31.19 | 125.61±15.95 | 19.27±2.71 | 205.12±109.11 | 31.48±4.07 | 520.54±73.87 | 167.45±15.67 | 814.76±49.81 |
| **300** | **4** | ND | 471.01±62.13 | 1975.89±65.36 | 970.79±55.13 | 215.11±85.86 | 188.05±25.86 | ND | ND | 408.04±11.39 | ND | 255.00±40.46 |
|  | **8** | ND | 503.31±57.16 | 2328.12±5.90 | 1039.08±30.62 | 239.57±63.08 | 76.10±84.40 | 274.69±15.50 | 199.78±2.27 | 613.56±0.79 | 126.46±5.68 | 93.47±19.04 |
|  | **12** | ND | 625.29±39.43 | 2667.04±109.95 | 1142.21±43.14 | 302.54±58.44 | 140.52±20.16 | 289.31±31.46 | 6.74±1.02 | 696.72±43.90 | 145.19±5.64 | 88.15±42.32 |
|  | **24** | ND | 1062.40±137.28 | 2753.98±4.17 | 729.17±56.82 | 315.72±76.74 | 12.54±4.10 | 80.49±2.17 | 6.68±2.49 | 480.55±10.85 | 152.91±5.87 | 115.58±5.26 |

ND = Not detected

arabinose = A1, xylose = X1, xylobiose = X2, xylotriose = X3, xylotetraose = X4, xylopentaose = X5, xylohexaose =X6, 2^3^-α-L-arabinofuranosyl-xylotriose = A2XX, 3^2^-α-L-arabinofuranosyl-xylobiose = A3X, 3^3^-α-L-arabinofuranosyl-xylotetraose = XA3XX, and 2^3^,3^3^-di-α-L-arabinofuranosyl-xylotriose = A2,3XX

**Supplementary Table S2**

Quantitative analysis of XOS and AXOS produced by Ultraflo Max

| **Enzyme**  **Conc. (U/g substrate)** | **Time (h)** | **Sugar content (ppm)** | | | | | | | | | | |
| --- | --- | --- | --- | --- | --- | --- | --- | --- | --- | --- | --- | --- |
|  |  | **A1** | **X1** | **X2** | **X3** | **X4** | **X5** | **X6** | **A2XX** | **A3X** | **XA3XX** | **A2,3XX** |
| **50** | **4** | ND | 371.76±48.39 | 1304.63±51.94 | 1240.64±166.41 | 768.46±197.67 | 258.16±7.10 | 102.16±6.38 | 164.81±9.13 | ND | 178.66±15.48 | ND |
|  | **8** | ND | 442.67±108.61 | 2205.19±17.52 | 1288.40±38.04 | 594.23±44.81 | 330.27±29.18 | 747.61±112.47 | 670.72±34.10 | ND | 590.46±1.03 | ND |
|  | **12** | ND | 1080.15±61.97 | 2096.23±71.36 | 1030.57±34.55 | 143.22±1.17 | 91.77±27.82 | 254.99±70.71 | 471.47±122.43 | ND | 219.92±21.08 | ND |
|  | **24** | ND | 2328.09±75.68 | 2647.98±172.41 | 1360.16±331.21 | 102.40±28.81 | 43.17±17.94 | 425.49±50.29 | 612.00±18.54 | ND | 496.87±18.40 | ND |
| **150** | **4** | ND | 1453.89±4.04 | 1873.30±88.55 | 741.79±29.91 | 123.64±26.67 | 76.08±8.14 | 281.07±39.30 | 375.90±56.63 | ND | 339.26±42.87 | ND |
|  | **8** | ND | 2373.37±75.68 | 1625.71±172.41 | 575.75±331.21 | 86.84±28.81 | 63.67±17.94 | 429.57±50.29 | 448.00±18.54 | ND | 519.42±18.40 | ND |
|  | **12** | ND | 2932.30±98.17 | 1404.91±84.41 | 448.02±36.31 | 82.28±12.30 | 47.76±24.43 | 184.21±2.21 | 432.45±9.35 | ND | 461.03±40.68 | ND |
|  | **24** | ND | 3712.00±60.52 | 924.97±17.10 | 229.49±22.15 | 80.04±15.94 | 21.59±16.07 | 167.31±77.63 | 430.25±91.75 | ND | 546.54±2.55 | ND |
| **300** | **4** | ND | 2385.83±85.21 | 1388.34±39.37 | 454.83±46.22 | 143.26±1.11 | 90.55±4.14 | 197.14±42.56 | 421.34±11.90 | ND | 356.99±51.86 | ND |
|  | **8** | ND | 2972.97±93.46 | 936.41±81.30 | 307.97±2.59 | 121.49±7.19 | 75.49±5.48 | 181.75±5.09 | 432.50±3.01 | ND | 374.30±115.50 | ND |
|  | **12** | ND | 3471.56±44.91 | 578.72±156.91 | 141.74±68.04 | 90.53±13.14 | 64.49±10.05 | 179.60±9.85 | 428.69±17.49 | ND | 477.37±48.51 | ND |
|  | **24** | ND | 3992.56±186.00 | 318.95±79.78 | 112.09±40.49 | 20.66±16.80 | 25.34±13.91 | 87.75±102.59 | 410.64±95.24 | ND | 479.50±64.84 | ND |

ND = Not detected

arabinose = A1, xylose = X1, xylobiose = X2, xylotriose = X3, xylotetraose = X4, xylopentaose = X5, xylohexaose =X6, 2^3^-α-L-arabinofuranosyl-xylotriose = A2XX, 3^2^-α-L-arabinofuranosyl-xylobiose = A3X, 3^3^-α-L-arabinofuranosyl-xylotetraose = XA3XX, and 2^3^,3^3^-di-α-L-arabinofuranosyl-xylotriose = A2,3XX
